# The modulating role of sources of difficulty in interactive matchstick algebra

**DOI:** 10.3389/fpsyg.2025.1691864

**Published:** 2026-01-19

**Authors:** Vladimir Spiridonov, Maria Erofeeva, Nils Klowait, Maxim Morozov, Vladlen Ardislamov

**Affiliations:** 1 Cognitive Research Lab, Faculty of Psychology, Institute for Social Sciences, Russian Presidential Academy of National Economy and Public Administration (RANEPA), Moscow, Russia; 2 Laboratory of Anthropology of Contemporary Worlds, Faculty of Philosophy and Social Sciences, Institute of Sociology, Université Libre de Bruxelles (ULB), Brussels, Belgium; 3 Transregional Collaborative Research Centre 318, Department of Technology and Diversity, Faculty of Mechanical Engineering, Paderborn University, Paderborn, Germany

**Keywords:** cognitive constraints, insight problem solving, interactivity, motor activity, perceptual chunks

## Abstract

Interactive problem solving has been proposed as an experimental manipulation that significantly increases the success of solving various matchstick algebra problems by allowing solvers to interact with physical representations of the problems. In contrast to this claim, we hypothesized that the influence of interactivity would vary based on the specific sources of difficulty inherent in the problems: perceptual chunks and cognitive constraints. We carried out a conceptual extended replication across three experimental series with conditions of varying degrees of interactivity, but failed to reproduce interactive solutions amongst our participants. A follow-up analysis of motor activity showed that the movements of the solvers did not contribute to chunk decomposition but significantly interfered with the relaxation of higher-level constraints. These findings suggest that motor activity can hinder performance when it does not align with the cognitive demands of the task. We therefore call for a more targeted and problem-specific understanding of how physical interaction contributes to restructuring in insight problem solving.

## 1 Introduction

When we encounter problems, we form their mental representations (Newell and Simon, 1972). Solving a problem may involve shifting or transforming the interpretation of the elements of a problem in a way that reveals a solution path. Restructuring refers to a mental process that changes how a problem is represented in the mind of the problem solver (Ohlsson, 1984; Wiley and Danek, 2024). The restructuring process can occur either through deliberate, conscious analysis of the problem’s structure, or through unconscious processes (as in spontaneous insight) (Öllinger et al., 2013; Fleck and Weisberg, 2013; Bilalić et al., 2019). It may involve recoding problem elements to see them differently, reinterpreting the goal or constraints of the problem, and relaxing assumptions that unnecessarily constrain thinking. Restructuring can be data-driven or conceptually driven (Weisberg, 2015; Korovkin et al., 2020): the former arises from perceptual changes in the problem’s representation, including its physical layout (Vallée-Tourangeau, 2025), while the latter results from deliberate internal analysis and reasoning about the problem.

Insight problems are designed so that a solution path is obscured or inaccessible. In insight problem solving, the solution appears suddenly–and often unexpectedly–following a shift in understanding and is often accompanied by an ‘aha moment’ (Wiley and Danek, 2024). Knoblich et al. (1999) designed insight matchstick algebra problems–initially false equations composed from Roman numerals–to put forward hypotheses about their relative difficulty: the solution of such problems is contingent upon overcoming two sources of difficulty. The solver has to move one matchstick in such a way that the equation becomes true. For example, to solve the statement ‘III = V + III’, the participant must move the vertical stick from the plus sign and place it next to the Roman numeral V, as to yield ‘III = VI − III’. Solvers have previous knowledge and assumptions about affordable arithmetic operations (constraints) and an ‘inherent’ integrity of numerals and operators (perceptual chunks) that mask the solution. Accordingly, finding the correct solution involves the decomposition of these chunks and the relaxation of some of the constraints. Through these processes, mental restructuring is achieved, which subsequently leads to insight.

Vallée-Tourangeau (2014) criticized the idea of mental restructuring as a form of methodological sequestering, the decoupling of the solver from their material environment. Restructuring is never purely mental, and the boundary between conceptually driven and data-driven restructuring is fuzzy: “It is […] striking to note how the analyses reported in Knoblich et al. (1999) […] ignore interactivity and its central role in thinking” (Vallée-Tourangeau, 2014: 35). In more recent studies of this research group (Vallée-Tourangeau et al., 2015, 2016a,b; Henok et al., 2020; Ross and Vallée-Tourangeau, 2020), interactivity is considered to be a condition through which new ideas emerge via active engagement with the material world. In practical terms, it is an experimental manipulation that varies the ability of the solver to interact with material artifacts. In contrast to a static condition, the interactive condition allows the solver to interact with physical objects that make up the components of the problem. In an earlier study involving the interactive condition (Weller et al., 2011), the authors used matchstick algebra problems and reported some major changes in success rates for problems of varying difficulty. It has been shown that interactivity essentially trumps the relative difficulty of the problems. Later experiments have shown that an interactive solution usually leads to an increase in the success rate for different types of problems (Vallée-Tourangeau et al., 2015, 2016a,b; Henok et al., 2020).

These results call for the fundamental reconceptualization of thinking processes (see Cowley, 2017; Ball, 2024); however, interactivity is an ill-defined and broad-brush term. It points to the role of the material representation of the problem, but at the same time hides concrete problem-solving mechanisms under the umbrella of a singular experimental manipulation. In this paper, we distinguished between the *interactive condition* (pertaining to the interactive experimental environment) and the *interactive solution* to stress that participants solve a problem with their hands. We operationalized “interactivity” in the following ways: (i) an interactive condition is an environment in which participants can physically manipulate the problem representation (e.g., matchsticks) as opposed to a static environment; and (ii) an interactive solution is a trial in which at least one coded movement occurs prior to an outcome. We seek to uncover the connection between the effects of interactivity, both as condition and solution, on performance and task difficulty. In (Weller et al., 2011), the interactive condition and solution have been conflated, and the greatest impact of interactivity has been observed for the most difficult types of problems (B, C, and D, but not A, see below). These results point to a potential link between task complexity and the effectiveness of interactive solutions.

In the next section, we introduce matchstick algebra problems in greater detail and provide conceptual arguments in favor of the claim that interactivity will impact performance on different types of problems unequally.

## 2 Matchstick algebra

In examining matchstick algebra problems, Knoblich et al. (1999) posited that the visual system delineates them into a tri-level representation: numerals (e.g., I, II, III); functional terms (e.g., I + V, III − II); and entire equations (e.g., VI = V + I). These levels embody a hierarchy where a modification at a higher level culminates in a more expansive revision of the ensuing problem representation. Aligning with this hierarchical structure, three distinct constraints are mapped to each representational level. Specifically, “(a) The value constraint applies at the level of numerals; (b) the operator constraint applies at the level of functional terms; and (c) the tautology constraint applies to changes that transform the structure of an entire equation” (Knoblich et al., 1999, p. 1537).

Moreover, numerals can be categorized into chunks, representing both composite and single-unit structures. Composite numerals like II, IV, VIII, and XI are designated as loose chunks, wherein, despite their recognition as singular entities representing numerical values, they are concurrently perceived as assemblies of individual symbols or smaller chunks (e.g., VII is perceived as V, I, and I). Contrarily, numerals like I, V, X, and the minus sign are recognized as tight chunks, perceived predominantly as indivisible units, with their decomposition into separate lines being seldom useful or meaningful. The plus and equal signs embody an intermediate chunk category; they disintegrate into potentially meaningful components, though such decompositions are rarely acknowledged or utilized.

**Figure 1:**
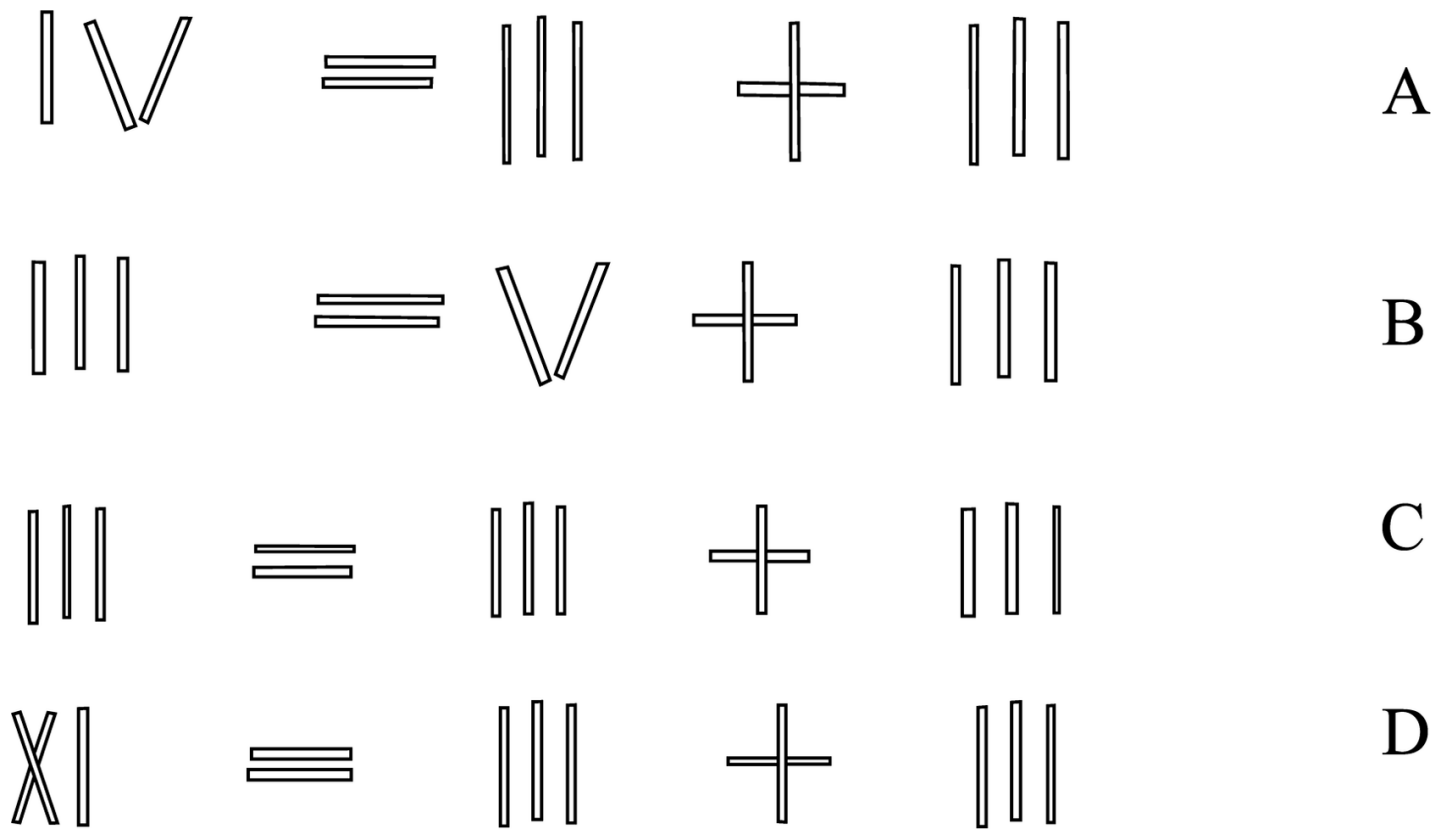
Problems of matchstick algebra.

A problem of type A (see Figure 1) involves the relaxation of the value constraint (‘the Roman numeral in the false equation can be changed only through an arithmetic operation’) and the decomposition of loose chunks (one has to ‘tear off’ ‘I’ from ‘IV’ and move it to another place). A problem of type B involves the relaxation of the operator constraint (‘the sign of an arithmetic operation cannot be changed’) in addition to the value constraint and decomposing the loose and intermediate chunks (one has to ‘tear off’ ‘I’ from ‘+’ and transfer it to a numeral). A type C problem involves relaxing the tautology constraint (‘the form of the equation should remain unchanged’) and decomposing the intermediate chunks (one has to turn the ‘+’ sign into a ‘=’ sign by moving one matchstick). A problem of type D involves the relaxation of the value constraint and the decomposition of tight chunks (one has to move one matchstick to turn ‘X’ into ‘V’). In general, Knoblich et al. (1999) predicted that “constraints are more difficult to relax the higher the level at which they apply” (p. 1537), i.e., the likelihood of relaxing the tautology constraint is lower than that of the operator constraint, which is also less likely to be relaxed than the value constraint. Furthermore, the authors posited that the tighter the chunks, the lower the probability of their decomposition.

Based on these theoretical assumptions, Knoblich et al. (1999) formulated two hypotheses. First, a problem of type A is easier to solve than a problem of type B, which in turn is less difficult than a problem of type C. This hypothesis translates into three predictions: A will be easier than B; A will be easier than C; B will be easier than C. Second, a type D problem is more difficult to solve than a type A problem. Thus, the difficulty of the task is dependent on particular moves that are required to solve the problem (whether tight chunks need to be decomposed, as in problem type D, or whether the constraint of higher levels needs to be relaxed, as in problem type C). These hypotheses have been confirmed experimentally (Knoblich et al., 1999).

### 2.1 Matchstick algebra and interactivity

Weller et al. (2011) set out to juxtapose the cognitive processes of insight problem solving described by Knoblich et al. (1999) in two distinct experimental setups—static and interactive. The interactive condition and solution had been conflated in that study. The static group encountered a two-dimensional representation of the false algebraic expression, while the interactive group interacted with a three-dimensional version. Weller et al. (2011) reported that Knoblich et al.’s (1999) predictions replicated only in the static condition. In the interactive condition, some major changes in the pattern in the mean percentage success for each of the four types of problems were observed: type A and B problems—as well as type A and D problems, respectively—had almost identical solution rates. The most difficult problem, type C, was still solved significantly less frequently than type B, although the success rate was 2.5 times higher in the interactive condition. Their results suggest that the effects of interactivity ‘neutralize’ the effects of the relative difficulty of the tasks discussed by Knoblich et al. (1999).

Nevertheless, some patterns in their results speak to the fact that this connection also functions in reverse: the effects of interactivity were most pronounced in the most difficult types of problems C and D (the success rates were more than 2 times higher for both). Moreover, type A problems were solved more successfully in the static condition; in other words, there were no interactivity effects for the simplest problem type.

A recent exploratory study of Vallée-Tourangeau (2025) employed matchstick algebra problems of types A and B and showed general performance improvement in the interactive condition. Again, the most pronounced effect was observed for a more complicated Problem B, and the analysis of solution processes revealed that in the interactive condition, physical restructuring of a problem precedes mental restructuring (termed “outsight”) in more than half of the cases.

One possible explanation for these patterns is that movements have uneven relevance depending on the source of problem difficulty. The source of difficulty may promote a preference for non-interactive (i.e., mental) solutions. In the simplest type A problem, it may be easier to rely on conceptually driven restructuring. In support of this claim, we can note Kirsh’s (2013) study on the process of learning a new dance phrase. The author shows that—in simple tasks—it is easier for practitioners to rely on mental images, while learning a complex dance move involves the embodied creation of a simplified version of the process. Although the context of his study is more general, we can draw from it the idea that the need for ‘thinking with things’ arises from the relative difficulty of the problem itself.

Relaxing different types of constraints and decomposing perceptual chunks may require different types of movements from the solver, as they may result in more or less relevant changes to the problem representation. For example, it is quite possible to imagine variants of movements aimed at decomposing a chunk in tasks A and D. When participants are separating the material representation of a numeral into individual sticks with their hands, they are physically decomposing the respective chunks. Yet it is difficult to suggest a type of solver movement that would relax the tautology constraint and that would “hint at” a different form of the equation, thereby increasing the success of solving problem C. Physical movements appeal to the specific properties of the elements of the problem rather than to abstractions such as the form of the equation.

## 3 The account of a failed replication

This paper is an extension of our previous work (Spiridonov et al., 2021), where we have undertaken a conceptual replication of the cited experiment (Weller et al., 2011). We intended to test whether a singular experimental manipulation (interactivity) can increase the success rate of solving matchstick algebra problems with different sources of difficulty. The procedure for the interactive group was identical to Weller et al. (2011) except for the time provided for each problem: we chose a 5-min rather than a 3-min interval in accordance with Knoblich et al. (1999).

In short, we have not been able to replicate the original experiment since our interactive condition did not prompt an interactive solution. For instance, in Experiment 1, the average number of movements was the following: task A—0.56; B—1.70; C—1.91; D—8.26 (see Table 1). In order to ascertain that our interactive conditions were indeed interactive, we employed specific experimental manipulations and participant instructions (Exp. 2) as well as an additional experimental run in a custom VR environment (Exp. 3). Bearing in mind that an interactive condition does not automatically lead to an interactive solution, we attempted to create conditions that afforded interaction with the physical objects. In total, in our conceptual replication, we designed and conducted three series of experiments. Experiment 1 was an initial replication of the effect of interactivity. In Experiments 2 and 3, we had extended the original replication by constructing experimental settings with arguably increased degrees of interactivity to compare their influence on the performance and the number of movements of the solvers. Unfortunately, our attempt to encourage participants to solve problems with their hands by creating experimental environments of various degrees of interactivity in Exp. 2 and 3 was unsuccessful. The total number of movements in all of our experiments was relatively small (see Table 1). This suggests that participants rarely interacted with the physical representation of the problem, and we could not draw any conclusions about the effects of the interactive solution.

**Table 1:** Descriptive statistics for all movements (excluding the final move) in Problems A, B, C, D in Experiments 1–3.

Experiment number	Statistical indicators	A	B	C	D
Experiment 1 (*n* = 54)	Mean	0.56	1.70	1.91	8.26
	Median	0	0	0	7
	Std. deviation	1.298	3.057	3.416	7.080
	Minimum	0	0	0	0
	Maximum	6	13	17	34
	Total amount	30	92	103	446
Experiment 2 (*n* = 45)	Mean	0.63	2.80	4.29	13.51
	Median	0	1	1	7
	Std. deviation	2.083	4.550	8.761	15.978
	Minimum	0	0	0	2
	Maximum	11	19	51	90
	Total amount	26	115	176	554
Experiment 3 (*n* = 45)	Mean	0.54	1.74	1.85	1.17
	Median	0	0.5	1	0
	Std. deviation	1.223	3.151	2.666	2.694
	Minimum	0	0	0	0
	Maximum	7	17	14	14
	Total amount	25	80	85	54
Experiments 1–3 (*n* = 144)	Mean	0.57	2.04	2.58	7.48
	Median	0	0	1	5
	Std. deviation	1.536	3.590	5.463	10.872
	Minimum	0	0	0	0
	Maximum	11	19	51	90
	Total amount	81	287	364	1,054

In this paper, we build on these results to study how two factors–the sources of difficulty of the problems and the motor activity of the solvers–influence the performance on matchstick algebra problems. We propose that physical interaction (i.e., motor activity) primarily contributes to data-driven restructuring, in which perceptual engagement with the material form of the problem prompts representational change. This kind of restructuring is more relevant when the difficulty lies in perceptual chunk decomposition (as in Problems A and D), where physical manipulation can directly alter the problem representation and support insight. In contrast, conceptually driven restructuring, which involves abstract reanalysis and constraint relaxation (as in Problems B and C), is less likely to be supported by motor activity, as the critical representational shift cannot be easily cued by the material form of the problem. We therefore hypothesize that the number of movements solvers perform affects the success of solving problems with different sources of difficulty unequally:

1. The lower the level at which the constraints apply and the greater the number of movements solvers perform, the higher the success rate.
2. No matter how tight the chunks are, the greater the number of movements solvers perform, the higher the success rate.

Based on these hypotheses, we predict that with a greater amount of motor activity: (1) A will be easier than B; A will be easier than C; B will be easier than C; (2) A will not be easier than D.

These hypotheses follow from the idea that data-driven restructuring, supported by motor activity, should benefit problems that require the decomposition of perceptual chunks. At the same time, the relaxation of constraints seems to be less affected by the physical representation of the problem; therefore, the pattern of results should not change in comparison to the original experiment of Knoblich et al. (1999).

Before we report the results of our current analysis that inform the contribution of this paper (4.4.4), we describe the experimental procedures used (4.1–4.3) and report the results of our previous unsuccessful replication experiments (4.4.1–4.4.3).

## 4 Experiments 1–3. Conceptual replication of the effect of interactivity in various interactive conditions

### 4.1 Interactivity operationalization

Following our previous work (Spiridonov et al., 2021), we distinguish between the interactive condition and the interactive solution. In this paper, we operationalized “interactivity” in the following ways: (i) an *interactive condition* is an environment in which participants can physically manipulate the problem representation (e.g., matchsticks, pencils, VR objects) as opposed to a static environment; and (ii) an *interactive solution* is a trial in which at least one coded movement occurs prior to an outcome. Since there is currently no consensus on the factors at play in the decision to solve problems with hands or in the head, we were only able to vary the interactivity of the condition (rather than the solution).

### 4.2 Participants, materials, and procedures

Across all experiments, participants were individually tested on Roman numeral recognition prior to problem solving by naming numerals from I to XII written by the experimenter in a random order; those who made more than two errors were excluded from participation. All participants were university students who gave written informed consent in accordance with the Declaration of Helsinki (World Medical Association, 2013) and received course credit for their participation.

Problem sets, timing, and randomization procedures were consistent throughout. The problem-solving task consisted of four matchstick algebra problems adapted from Knoblich et al. (1999) (see Figure 1), with one version of each problem type (A, B, C, and D). The task instruction was: “You need to move one stick so that the equation is correct.” It was given verbally before each trial and repeated if prompted; in addition, a written version of the instruction was placed within the participant’s view. In all experiments, 5 min were allotted for solving each problem, as in the original Knoblich et al. (1999) study. Problems not solved within that window were considered unsolved. The problem order was randomized using a Latin square.

For the sake of clarity, we henceforth use uniform labels for different experimental conditions across our three experiments: ‘static’ and ‘interactive’ will denote the interactivity of the environment, ‘paper’ and ‘stick’ will indicate the type of material used, and the addition of ‘assembled’ means that participants have to recreate an equation from a presented photograph prior to solving it. In all interactive conditions across the three experiments, participants were instructed: “Please move the sticks—the problem can only be successfully solved if you move the sticks with your hands.” Additionally, following a set protocol, the experimenter reminded participants to use their hands every 90 s.

Experiment 1 was set up to replicate the basic interactivity effect using physical materials. 108 participants took part in Experiment 1 (*M* = 19.6; *SD* = 1.84; 79% female). Three participants were not allowed to take part in the experiment due to low Roman numeral recognition. The final *N* = 105.

Participants were randomly assigned to a *Static Sticks* condition, where problems were preassembled and solved verbally without touching the materials, or an *Interactive Assembled Sticks* condition, where participants recreated and solved the problems using magnetic matchsticks on a board. Solving time began once the problem was fully recreated (interactive) or when prompted (static).

Experiment 2 extended the design by introducing four experimental conditions varying in interactivity and materiality. 90 participants took part in Experiment 2 (*M* = 19.8; *SD* = 2.40; 81% female). Two participants were not allowed to take part in the Experiment due to low Roman numeral recognition. The final *N* = 88.

Participants were randomly assigned to a *Static Paper* (photograph of sticks, no physical interaction), *Static Sticks* (as in Experiment 1), *Interactive Sticks* (participants manipulated preassembled problems), and *Interactive Assembled Sticks* (participants assembled and solved the problem). Unsharpened pencils replaced plastic matchsticks to enhance motor usability.

Experiment 3 (*N* = 74; *М* = 19.7; *SD* = 1.56; 79% female) employed another strategy to enhance interactivity by transitioning to a virtual reality (VR) environment that allowed us to simulate sensorimotor representations of various material environments. One participant was not allowed to take part in the Experiment due to low Roman numeral recognition. The final *N* = 73. Participants had no prior VR experience.

With VR, we intended to create an experimental environment in which only relevant affordances are actualized by creating a maximally sparse experimental environment with minimal distractions. Participants were divided into three VR conditions: *Static VR* (no interaction, verbal responses only), *VR Interactive Sticks* (manipulation of preassembled problems), and *VR Interactive Assembled Sticks* (participants assembled and solved problems in VR). The VR environment was created using the Unity Game Engine; an HTC Vive Pro and Meta Quest, enabling motion tracking in six degrees of freedom, were used. Interactions were mediated by a VR headset and a hand-held controller, enabling precise manipulation of virtual matchsticks. In both interactive VR conditions, participants could reach toward a stick, highlight it, press a button to grasp it, and freely move, rotate, or release it in 3D space. We implemented a training activity before problem solving to familiarize participants with VR equipment and controls.

### 4.3 Movement coding

To control that our interactive conditions were indeed interactive, we quantified participants’ epistemic manipulation during problem solving by coding the number of movements executed throughout the solution process.

To identify these movements, we analyzed video recordings from the experiments. At a preliminary stage, five co-authors collaboratively developed a movement taxonomy following Kirsh and Maglio (1994), Kirsh (2010), and Vallée-Tourangeau et al. (2015). Through group discussions, we identified several types of actions involving the sticks during the 5-min solving interval. The resulting coding scheme (see Table 2) allowed us to capture a range of distinct task-relevant motor activities in relation to the problem representation with appropriate granularity.

**Table 2:** Movement coding scheme of a participant’s manipulation of matchsticks, pencils and virtual sticks across three experiments.

Type number	Description	Example
1. Markings	The solver performs a motion with the pencil as a result of which the appearance of the task does not change significantly, i.e., the pencil does not change its location. An indexical gesture is considered marking only if the distance between the fingers of the solver and the pencil does not exceed 2 cm.	The solver grasped the pencil with their fingers but did not move the pencil to a new location.
2. Pragmatic actions	The solver takes the pencil and moves it to a new place in the equation, resulting in a possible solution. The trajectory of the movement should be smooth and continuous. By the end of the pragmatic action, the solver will have placed the pencil in a new place, either releasing it or holding it in this new place for over a second. Notable pauses in the process of performing the movement disqualify the action from being marked as pragmatic.	The solver takes the pencil and—in one uninterrupted motion—places it in some other spot in the equation.
3. Epistemic actions	An action with a pencil that changes the appearance of the task significantly, yet does not result in a possible solution, since the pencil is not transferred to a new place in the equation.	The solver takes a pencil and manipulates it in the air, and then returns it to its original location.
4. A tie of epistemic and pragmatic actions	Two or more successive movements are made with the same pencil. That is, the participant does not release the pencil with which the actions are performed. At least one movement in this chain must meet the criteria of an epistemic action.	The solver takes a pencil and—in a wide arc, suspending the motion several times in the air—slowly transfers the pencil to a new place. Then the participant takes their hand off the pencil.
5. A tie of marking and pragmatic actions	These are actions corresponding to the criteria of marking and pragmatic action performed with the same pencil without interruption in time.	The solver touches the pencil, holds it in their fingers without taking it off the table, then moves it to a new place in the equation.
6. A tie of marking and epistemic actions	Actions that meet the criteria for marking and epistemic action are performed with the same pencil without interruption in time.	The solver touches the pencil, twists it a little without taking it off the table, then, without releasing the pencil, picks it up, but immediately returns it to its original place.
7. Any action from the above classes and epistemic question	Any action from the classes described above, performed together with a question—addressed to the experimenter—about the task rules or the correctness of the attempted solving.	The solver takes a pragmatic action and asks the experimenter—“Is this move possible?”
8. Reset action	The solver returns the pencil to its original place in the equation after an incorrect solving attempt.	The solver returns the pencil to its original place in the equation after an incorrect solving attempt.

Subsequently, independent coders (three per experiment), blind to the hypotheses, applied this scheme to the recorded data after a training session with the co-authors, where examples of movements of each type were demonstrated. All discrepancies were resolved through iterative group discussions with repeated video reviews. We did not compute a formal inter-rater reliability metric, assuming that the procedure of collective discussion during protocol annotation ensured an acceptable level of consistency. We also adopted this strategy because the total number of participant movements recorded in the protocols was relatively small. After that, all movements were summarized in a general summation of task-relevant movements (for descriptive statistics, see Table 1).

In the present analyses, we include all movements in successful solution trials except the final one, which is purely pragmatic and happens after the problem is solved; therefore, it is irrelevant to the epistemic problem solving processes. Because the total number of observed movements was relatively low, we opted not to use aggregated measures such as movement density (i.e., moves per minute).

### 4.4 Results

Our failed replication of an interactive solution demonstrates that the relation between the interactive condition of an environment and the number of movements solvers perform is not straightforward. We conducted a thorough analysis of our data to understand the role of interactive conditions in matchstick algebra problem solving. We report combined results for all three experiments because the structure of the analysis was consistent throughout.

To investigate the effect of the interactive conditions (experiment), problem type, and total number of movements on the solution rate, we conducted a Bayesian logistic regression. We choose the Bayesian approach for several reasons. Firstly, it allows us to obtain evidence for the null hypothesis in the context of a conceptual replication of the interactivity effect (Weller et al., 2011, see also Chuderski et al., 2020; Ross and Vallée-Tourangeau, 2020). Secondly, the Bayesian approach suits our relatively small sample sizes per condition in experiments 2 and 3 (20–25 participants, respectively).

A Bayesian logistic regression was conducted using the rstanarm package (Goodrich et al., 2025), to examine the effects of interaction of Experiment (1,2,3) and group [*Static Sticks, Assembled Sticks* (in Exp. 1); *Static Paper, Static Sticks, Interactive Sticks, Interactive Assembled Sticks* (in Exp. 2); *Static VR, VR Interactive Sticks, VR Interactive Assembled Sticks* (in Exp. 3)], problem type (A, B, C, D), total number of movements participants did during the solution, and duration of the solution period (3 min, 5 min) on the probability of a correct solution. Weakly informative Normal (0, 2.5) priors were used for all regression coefficients. Four Markov chains were run with 2,000 iterations each. Convergence diagnostics indicated no issues (all *Ȓ* < 1.01; no divergent transitions). Here and throughout all subsequent analyses, we adopted weakly informative priors. Prior work on the matchstick algebra problems used in our study (e.g., Knoblich et al., 1999; Weller et al., 2011; Vallée-Tourangeau, 2025) has reported highly variable solution rates, rendering the expected baseline performance uncertain. Moreover, participants in the present study exhibited success rates that exceeded those typically observed in the literature, further motivating the use of priors that impose minimal informational constraints. As an intercept, we used the success rate in the *Static Sticks* condition in Experiment 1, corresponding to a baseline accuracy of approximately 86%. The posterior medians, 95% CI and ROPE (±0.2 log-odds) coefficients for all predictors you can see in Table 3.

**Table 3:** Median, CI, and ROPE (±0.2 log-odds) for the unified Bayesian regression model predictors.

Predictors	Median	95% CI	ROPE (±0.2 log-odds)
Intercept	1.81	[1.19, 2.42]	0%
*Assembled Sticks (Exp. 1)*	−0.1	[−0.43, 0.23]	71.4%
*Static Paper (Exp. 2)*	0.08	[−0.34, 0.49]	64%
*Static Sticks (Exp. 2)*	−0.04	[−0.49, 0.41]	65%
*Interactive Sticks (Exp. 2)*	0.17	[−0.27, 0.64]	53%
*Interactive Assembled Sticks (Exp. 2)*	0.11	[−0.32, 0.55]	61%
*Static VR (Exp. 3)*	0.28	[−0.17, 0.74]	36%
*VR Interactive Sticks (Exp. 3)*	0.67	[0.22, 1.15]	0%
*VR Interactive Assembled Sticks (Exp. 3)*	0.16	[−0.23, 0.58]	56%
Problem B	−1.59	[−2.07, −1.17]	0%
Problem C	−2.95	[−3.40, −2.54]	0%
Problem D	−1.93	[−2.40, −1.51]	0%
Total number of movements	−0.015	[−0.042, 0.014]	100%
Duration of the solution (5 min)	0.27	[0.16, 0.38]	8%

Across all posterior estimates for contrasts within experimental groups, only the *VR Interactive Sticks* condition showed clear evidence of an effect, corresponding to an accuracy of approximately 92% (+6 pp). All other posterior estimates for contrasts within all experimental groups showed substantial uncertainty. For all these contrasts, the 95% credible intervals included zero, and a considerable proportion of the posterior mass fell within the ROPE, suggesting little evidence of meaningful differences between these condition levels.

Three problem types contrasts showed large, negative, and precise effects. The accuracy for *Problem B* was approximately 55%, for *Problem C* − 24% and for *Problem D* − 57% indicating that participants performed worse on Problems B, C, and D than on task A. ROPE analysis (±0.2 log-odds) for all problem types showed 0% of the posterior within the ROPE, indicating a credible and practically non-zero effect. In contrast, the *Total number of movements* showed substantial uncertainty. At the same time, the duration of the solution period showed that more participants found a solution in the five-minute period than in the three-minute period.

Bridge sampling yielded a Bayes factor greatly exceeding 1,000 in favor of the full model over the baseline model without predictors, indicating overwhelming evidence that the predictors collectively improve predictive performance.

The model that included all predictors indicated that only one experimental group (*VR Interactive Sticks* condition in Experiment 3) showed credible evidence of a deviation from the baseline, as reflected by its posterior distribution. Experiment duration and problem type also exhibited robust effects. In contrast, the posterior for the total number of movements was centered near zero and showed substantial overlap with the baseline, providing little evidence that this predictor influenced the success rate.

To further examine the effect of interactivity on solution rate, we conducted separate Bayesian logistic regression analyses for each problem type within each experiment. This approach was motivated by the fact that different problem types are associated with distinct sources of difficulty (Knoblich et al., 1999). Accordingly, we expected that interactivity would influence solution rate differentially depending on the underlying source of difficulty.

#### 4.4.1 The effect of the interactive condition

##### 4.4.1.1 Five minute period

Descriptive statistics for the success rate in all three experiments are presented in Tables 4–6. A Bayesian logistic regression was used to investigate the effect of the interactive conditions on the solution rate. For all analyses, we used weakly informative priors on all coefficients [Normal (0, 2.5)] for the same reasons stated above. Four Markov chains were run for 2,000 iterations each, with 1,000 warmup iterations. Convergence diagnostics indicated no issues (all *Ȓ* < 1.01; no divergent transitions).

**Table 4:** Success rate in two experimental conditions in Experiment 1.

Condition	Problem type			
	A	В	С	D
	Proportion solved			
Interactive assembled sticks (*N* = 54)	0.91	0.87	0.59	0.67
Static sticks (*N* = 51)	0.94	0.80	0.59	0.69

**Table 5:** Success rate in four experimental conditions in Experiment 2.

Group	Problems			
	A	В	С	D
	Proportion solved			
Group 1 (*N* = 21) interactive sticks	1	0.76	0.52	1
Group 2 (*N* = 20) static sticks	1	0.85	0.35	0.90
Group 3 (*N* = 23) static paper	0.96	0.87	0.52	0.83
Group 4 (*N* = 24) interactive assembled sticks	1	0.88	0.63	0.67

**Table 6:** Success rate in three experimental conditions in VR in Experiment 3.

Condition	Problems			
	A	В	С	D
	Proportion solved			
Group 1 (*N* = 24) interactive sticks	1	0.92	0.62	0.96
Group 2 (*N* = 21) VR interactive assembled sticks	1	0.76	0.67	0.81
Group 3 (*N* = 28) static VR	1	0.86	0.57	0.82

We found that—for problem A—in Experiment 1, the posterior median for the intercept was 2.3 [95% CrI (1.5, 3.4)], corresponding to a baseline accuracy in the Static Sticks condition of approximately 91%. The effect of the *Assembled Sticks* condition was uncertain: the posterior median was 0.49 [95% CrI (−1.05, 2.08)], with Pr(*β* > 0) = 0.73. This corresponds to a predicted accuracy of approximately 94% (+2 pp), but with substantial uncertainty. The Bayes factor (BF₁₀ = 0.25) provided moderate evidence in favor of the null model relative to the model including condition. However, only 8% of the posterior distribution for the condition coefficient fell within the predefined ROPE (−0.2, 0.2 on the log-odds scale), indicating insufficient posterior mass to claim practical equivalence to zero. Taken together, the credible interval, Bayes factor, and ROPE analysis suggest that the data do not allow a firm conclusion about the presence or the practical absence of a condition effect.

In experiments 2 and 3, problem A was excluded from the analysis since all but one participant solved it successfully.

For problem B—in Experiment 1, the posterior median for the intercept was 1.9 [95% CrI (1.2, 2.81)], corresponding to a baseline accuracy in the *Static Sticks* condition of approximately 87%. The effect of the *Assembled Sticks* condition was uncertain: the posterior median was −0.49 [95% CrI (−1.61, 0.53)], with Pr(*β* > 0) = 0.175. This corresponds to a predicted accuracy of approximately 80% (−7 pp), but with substantial uncertainty. The Bayes factor (BF₁₀ = 0.3) provided moderate evidence in favor of the null model relative to the model including condition. However, only 11% of the posterior distribution for the condition coefficient fell within the predefined ROPE (−0.2, 0.2 on the log-odds scale), indicating insufficient posterior mass to claim practical equivalence to zero. Taken together, the credible interval, Bayes factor, and ROPE analysis suggest that the data do not allow a firm conclusion about the presence or the practical absence of a condition effect.

In Experiment 2, the posterior median for the intercept was 1.96 [95% CrI (0.97, 3.44)], corresponding to a baseline accuracy in the *Static paper* condition of approximately 87%. The effects of *Static Sticks, Interactive sticks* and *Interactive assembled sticks* were uncertain: the posterior median was −0.13 [95% CrI (−2, 1.71)], −0.76 [95% CrI (−2.49, 0.8)], 0.07 [95% CrI (−1.77, 1.9)], with Pr(*β* > 0) = 0.44, Pr(*β* > 0) = 0.17, Pr(*β* > 0) = 0.53, respectively. This corresponds to a predicted accuracy of approximately 86% (−1 pp), 77% (−10 pp), 88% (+1 pp), respectively, but with substantial uncertainty. The Bayes factor (BF₁₀ = 0.1) provided moderate evidence in favor of the null model relative to the model including condition. However, only 18, 13 and 20% of the posterior distribution for the condition coefficients, respectively, fell within the predefined ROPE (−0.2, 0.2 on the log-odds scale), indicating insufficient posterior mass to claim practical equivalence to zero. Taken together, the credible interval, Bayes factor, and ROPE analysis suggest that the data do not allow a firm conclusion about the presence or the practical absence of a condition effect.

In Experiment 3, the posterior median for the intercept was 1.84 (95% CrI [0.86, 3.12]), corresponding to a baseline accuracy in the *Static VR* condition of approximately 86%. The effects of *VR Interactive sticks* and *VR Interactive Assembled Sticks* were uncertain: the posterior median was 0.66 (95% CrI [−1.14, 2.68]) and −0.66 (95% CrI [−2.24, 0.8]), with Pr(*β* > 0) = 0.77 and Pr(β > 0) = 0.19, respectively. This corresponds to a predicted accuracy of approximately 92% (+6 pp) and 76% (−10 pp), respectively, but with substantial uncertainty. The Bayes factor (BF₁₀ = 0.26) provided moderate evidence in favor of the null model relative to the model including condition. However, only 14 and 16% of the posterior distribution for the condition coefficients, respectively, fell within the predefined ROPE (−0.2, 0.2 on the log-odds scale), indicating insufficient posterior mass to claim practical equivalence to zero. Taken together, the credible interval, Bayes factor, and ROPE analysis suggest that the data do not allow a firm conclusion about the presence or the practical absence of a condition effect.

For Problem С—in Experiment 1, the posterior median for the intercept was 0.29 [95% CrI (−0.21, 0.86)], corresponding to a baseline accuracy in the *Static Sticks* condition of approximately 57%. The effect of the *Assembled Sticks* condition was uncertain: the posterior median was 0.07 [95% CrI (−0.73, 0.82)], with Pr(*β* > 0) = 0.56. This corresponds to a predicted accuracy of approximately 59% (+2 pp), but with substantial uncertainty. The Bayes factor (BF₁₀ = 0.21) provided moderate evidence in favor of the null model relative to the model including condition. However, only 20% of the posterior distribution for the condition coefficient fell within the predefined ROPE (−0.2, 0.2 on the log-odds scale), indicating insufficient posterior mass to claim practical equivalence to zero. Taken together, the credible interval, Bayes factor, and ROPE analysis suggest that the data do not allow a firm conclusion about the presence or the practical absence of a condition effect.

In Experiment 2, the posterior median for the intercept was 0.09 [95% CrI (−0.75, 0.96)], corresponding to a baseline accuracy in the *Static paper* condition of approximately 52%. The effects of *Static Sticks*, *Interactive sticks* and *Interactive assembled sticks* were uncertain: the posterior median was −0.71 [95% CrI (−1.99, 0.49)], 0.01 [95% CrI (−1.2, 1.3)], 0.43 [95% CrI (−0.71, 1.64)], with Pr(*β* > 0) = 0.12, Pr(*β* > 0) = 0.51, Pr(*β* > 0) = 0.76, respectively. This corresponds to a predicted accuracy of approximately 35% (−17 pp), 52% (+0 pp), 63% (+11 pp), respectively, but with substantial uncertainty. The Bayes factor (BF₁₀ = 0.2) provided moderate evidence in favor of the null model relative to the model including condition. However, only 14, 27 and 22% of the posterior distribution for the condition coefficients, respectively, fell within the predefined ROPE (−0.2, 0.2 on the log-odds scale), indicating insufficient posterior mass to claim practical equivalence to zero. Taken together, the credible interval, Bayes factor, and ROPE analysis suggest that the data do not allow a firm conclusion about the presence or the practical absence of a condition effect.

In Experiment 3, the posterior median for the intercept was 0.29 [95% CrI (−0.45, 1.08)], corresponding to a baseline accuracy in the *Static VR* condition of approximately 57%. The effects of *VR Interactive sticks* and *VR Interactive Assembled Stick* were uncertain: the posterior median was 0.25 [95% CrI (−0.89, 0.42)] and 0.43 [95% CrI (−0.77, 1.66)], with Pr(*β* > 0) = 0.67 and Pr(*β* > 0) = 0.75, respectively. This corresponds to a predicted accuracy of approximately 63% (+6 pp) and 67% (+10 pp), respectively, but with substantial uncertainty. The Bayes factor (BF₁₀ = 0.26) provided moderate evidence in favor of the null model relative to the model including condition. However, only 27 and 21% of the posterior distribution for the condition coefficients, respectively, fell within the predefined ROPE (−0.2, 0.2 on the log-odds scale), indicating insufficient posterior mass to claim practical equivalence to zero. Taken together, the credible interval, Bayes factor, and ROPE analysis suggest that the data do not allow a firm conclusion about the presence or the practical absence of a condition effect.

For Problem D—in Experiment 1, the posterior median for the intercept was 0.69 [95% CrI (0.14, 1.28)], corresponding to a baseline accuracy in the *Static Sticks* condition of approximately 66%. The effect of the *Assembled Sticks* condition was uncertain: the posterior median was 0.09 [95% CrI (−0.73, 0.93)], with Pr(*β* > 0) = 0.59. This corresponds to a predicted accuracy of approximately 68% (+2 pp), but with substantial uncertainty. The Bayes factor (BF₁₀ = 0.21) provided moderate evidence in favor of the null model relative to the model including condition. However, only 19% of the posterior distribution for the condition coefficient fell within the predefined ROPE (−0.2, 0.2 on the log-odds scale), indicating insufficient posterior mass to claim practical equivalence to zero. Taken together, the credible interval, Bayes factor, and ROPE analysis suggest that the data do not allow a firm conclusion about the presence or the practical absence of a condition effect.

In Experiment 2, the posterior median for the intercept was 1.61 [95% CrI (0.66, 2.90)], corresponding to a baseline accuracy in the *Static paper* condition of approximately 83%. In contrast to all other comparisons, here we observe clear evidence for a positive effect of the *Interactive sticks* condition. The posterior median of the coefficient was 4.27 [95% CrI (0.59, 11.40)], with Pr(*β* > 0) = 0.99 and 0% of the posterior mass falling within the ROPE (−0.2, 0.2 on the log-odds scale), indicating strong evidence for a practically meaningful positive effect. This corresponds to a predicted accuracy of approximately 99% (+16 pp).

However, the effects of the *Static Sticks* and *Interactive assembled sticks* conditions were uncertain in line with all other comparisons: the posterior median was 0.66 [95% CrI (−1.08, 2.62)], −0.92 [95% CrI (−2.44, 0.41)], with Pr(*β* > 0) = 0.78, Pr(*β* > 0) = 0.09, respectively. This corresponds to a predicted accuracy of approximately 90% (+7 pp) and 67% (−6 pp), respectively, but with substantial uncertainty. However, only 14 and 10% of the posterior distribution for the condition coefficients, respectively, fell within the predefined ROPE (−0.2, 0.2 on the log-odds scale), indicating insufficient posterior mass to claim practical equivalence to zero. The Bayes factor (BF₁₀ = 2.86) provided only weak-to-moderate support in favor of the alternative model including condition, relative to the null model.

In Experiment 3, the posterior median for the intercept was 1.32 [95% CrI (0.48, 2.34)], corresponding to a baseline accuracy in the *Static VR* condition of approximately 79%. In contrast to all other comparisons, here we observe clear evidence for a positive effect of the *VR Interactive sticks* condition. The posterior median of the coefficient was 1.93 [95% CrI (0.03, 4.69)], with Pr(*β* > 0) = 0.97 and 2% of the posterior mass falling within the ROPE (−0.2, 0.2 on the log-odds scale), indicating that the effect is highly unlikely to be practically negligible. This corresponds to a predicted accuracy of approximately 96% (+17 pp). However, the effects of *VR Interactive Assembled Sticks* were uncertain: the posterior median was 0.16 [95% CrI (−1.28, 1.63)], with Pr(*β* > 0) = 0.77, Pr(*β* > 0) = 0.58 and 22% of the posterior mass falling within the ROPE (−0.2, 0.2 on the log-odds scale), indicating insufficient posterior mass to claim practical equivalence to zero. This corresponds to a predicted accuracy of approximately 81% (+2 pp). The model comparison revealed the Bayes factor (BF₁₀ = 0.43) provided weak evidence in favor of the null model relative to the model including condition. Taken together, the credible interval, Bayes factor, and ROPE analysis suggest that the data do not allow a firm conclusion about the presence or the practical absence of a condition effect.

##### 4.4.1.2 Three minute period

The three-minute interval analysis yielded results similar to those obtained in the five-minute analysis. Further details can be found in the Supplementary material.

#### 4.4.2 Summary

To summarize, we examined the effect of the interactive condition on solution rates using Bayesian logistic regression conducted separately for each problem type. In all but one case (Problem D in Experiment 2), the analyses yielded moderate or weak evidence in favor of the null model, which did not include the condition factor. This pattern suggests that we cannot confidently conclude that the interactive condition increases solution rates. At the same time, the evidence is not strong enough to assert that it has no effect.

#### 4.4.3 The influence of interactive conditions on success rates across the experiments

We have also conducted a combined analysis. To analyze the influence of our manipulations of the interactivity of the conditions we ran a binary logistic regression with Success rate as the dependent variable and Experiment (1, 2, 3) and Problem type (A, B, С, D) as predictors. We found that the odds of solving problem A were significantly higher at 860% [95% *CI* (0.07, 10.15)] *p* = 0.028 in Experiments 2 and 3 than in Experiment 1. However, the odds of solving Problems B and C were significantly lower in Experiments 2 and 3 than in Experiment 1 at 88% for both [95% *CI* (0.007, 0.55)] *p* = 0.034, [95% *CI* (0.007, 0.52)] *p* = 0.031, respectively. We did not find significant differences for Problem D across Experiments *p* = 0.12. The analysis suggests that the increase in interactivity leads to an increase in the solution rate only for Problem A and a decrease in the solution rate for Problems B and C.

This was the only result that indicated a relationship between the interactivity, performance, and sources of difficulty. However, as solvers did not move their hands much in our experiments, it is unclear what impacted the worsening of performance for problems with higher-level constraints.

Next, we present the results of our reanalysis where we focused on the motor activity of the solvers.

#### 4.4.4 Reanalysis: testing the effects of motor activity and sources of difficulty on problem solving in matchstick algebra

Without truly interactive solutions with a large amount of movement, we were unable to draw any conclusions about the effect of interactivity as a solution on performance. However, we videotaped and coded the motor activity of the solvers, which allowed us to test the hypotheses about the link between the number of movements of the solvers and the success rates for problems with different sources of difficulty. We expected that (1) motor activity would have a greater positive impact for lower levels of constraints; (2) positive effects of motor activity would be observed independent of the tightness of perceptual chunks.

##### 4.4.4.1 Comparison of the number of movements in Experiments 1–3

The descriptive statistics are provided in Table 1.

We ran a 3 × 4 repeated-measures ANOVA with the Problem (A, B, C, D) as a within-subject factor, and Experiment number (Experiment 1/Experiment 2/Experiment 3) as a between-subject factor. As a dependent variable, we used the total number of movements. The ANOVA revealed significant differences in the number of movements across the four problems *F*(3,414) = 64.231, *p* < 0.001, *ηp2* = 0.318. A *post hoc* pairwise comparison with a Bonferroni correction for multiple comparisons revealed that the number of movements was significantly higher in Experiment 2 in comparison to Experiment 3 (*p* < 0.001) and was not significantly different from Experiment 1. The interaction of factors was significant *F*(6,414) = 17.847, *p* < 0.001, *ηp2* = 0.206. A *post hoc* pairwise comparison with a Bonferroni correction for multiple comparisons revealed that the number of movements during problem solving was the following: D > C > B > A (*p* < 0.001 for all cases); С > A (*p* < 0.001); and B > A (*p* < 0.001).

As we have obtained a low number of movements in Experiments 1–3, we have decided to analyze the effect of movements on success rate using the combined results of all 3 experiments. This would allow us to capture smaller effects and decrease the probability of false-negative conclusions compared to a separate analysis.

##### 4.4.4.2 Comparison of the number of movements across the correct and incorrect solutions in Experiments 1–3

We ran a 3 × 2 ANOVA with Experiment number (Experiment 1/Experiment 2/Experiment 3) and correctness of solution (0/1) as between-subject factors. As a dependent variable, we used the total number of movements. The descriptive statistics are provided in Table 1. The ANOVA revealed significant differences in the number of movements across the correct and incorrect solutions for Problems B *F*(1,140) = 11.264, *p* = 0.001, *ηp2* = 0.077, and C *F*(1,140) = 5.211, *p* = 0.024, *ηp2* = 0.037. In both cases, the mean number of movements was significantly higher for unsuccessful attempts compared to correct solutions for problems B and C. Contrary to Hypothesis 1, motor activity did not have a greater positive impact on lower-level constraints; in fact, it had a significant negative impact on higher-level constraints. Furthermore, for problems C and D, there was a significant difference in the number of movements in the three Experiments *F*(2,140) = 3.333, *p* = 0.039, *ηp2* = 0.047, and *F*(2,140) = 5.555, *p* = 0.005, *ηp2* = 0.076, correspondingly. A *post hoc* pairwise comparison with a Bonferroni correction for multiple comparisons revealed that the number of movements was significantly higher in Experiment 2 in comparison to Experiment 3 (for problem C *p* = 0.05, for problem D *p* = 0.004), and was not significantly different from Experiment 1. The interaction of factors was non-significant for all cases.

#### 4.4.5 Discussion

In all three experimental paradigms we used, a similar pattern of results was obtained: we did not observe an effect of the interactive condition on the success of solving matchstick algebra problems, and only the problem type influenced the success of the solution. The structure of the results obtained was comparable to the results of Knoblich et al. (1999). In Experiment 2, we fully reproduced Knoblich et al.’s (1999) findings (success in solving problem A > success in solving problem B > success in solving problem C; and success in solving problem A > success in solving problem D). We reproduced the findings to a significant extent (three of its four predictions) in Experiment 1, and to some extent (two out of four) in Experiment 3.

These results are not surprising since we were not able to solicit interactive solutions from our participants. The modest number of movements performed by our experiment participants indicates that the creation of interactive conditions is insufficient for inducing interactive solutions among the participants.

In our reanalyses, we found a significantly greater number of movements across participants in Experiment 2 compared to Experiment 3. It also turned out that the total number of movements is not related to the success of solving problems A and D and is a predictor of the unsuccessful solution of problems B and C. Recall that the successful solution of problems A and D is associated with the decomposition of the perceptual chunks, problem B is associated with overcoming the operator constraint, and problem C is associated with the tautology constraint (Knoblich et al., 1999). Thus, it turned out that the movements of the solvers do not affect the decomposition of chunks but significantly interfere with overcoming two higher-level types of constraints: operator and tautology.

These results do not confirm our hypotheses, but nevertheless align with them. We predicted that the greater number of movements would (1) affect the success rate for problems A, B and C, but the constraints inherent in such problems would not be affected; (2) increase the success rate for problems A and D with differing chunk tightness. Our results show an interaction of the sources of difficulty of the problem and the number of movements of the solvers, but opposite to what we expected. Perceptual chunks are not affected by solver movements, no matter their tightness, while higher-level constraints are negatively affected by the motor activity.

## 5 General discussion

### 5.1 From interactive condition to interactive solution

In interactivity research (Vallée-Tourangeau et al., 2015, 2016a,b; Vallée-Tourangeau and Vallée-Tourangeau, 2017; Henok et al., 2020; Ross and Vallée-Tourangeau, 2020), it was assumed that creating a possibility to manipulate physical objects during problem solving (the so-called interactive condition) would lead to an increase in the participants’ performance since the affordances of material objects would reduce cognitive load, provide new perceptual information, and help restructure the problem representation. We cast doubt on the idea that interactivity could equally affect problems that are characterized by different sources of difficulty and therefore explored the interplay between the relative difficulty of the mental tasks and interactivity, hypothesizing that the effects of interactivity could potentially vary across different mental constraints on the problem representation and perceptual chunks.

In our three experimental series, we intended to use the interactivity of the condition as the facilitator of solutions. After an unsuccessful Experiment 1, we sought to increase the interactivity of the conditions through specific experimental manipulations and participant instructions (Exp. 2) as well as a custom VR environment (Exp. 3). Notwithstanding our manipulations, the total number of solver movements remained low across our experiments, and we thus observed that providing an interactive condition does not necessarily elicit interactive solutions. This finding is theoretically meaningful: it stresses the idea that merely rendering a condition interactive does not instantaneously translate to an increase in the number of movements or a higher success rate. Other studies also indicate that the mere presence of a physical representation of a problem does not contribute to a successful solution in all cases (Chuderski et al., 2020; Ross and Vallée-Tourangeau, 2020, Spiridonov et al., 2021, Wang and van Leeuwen, 2022). It becomes evident that interactivity needs to be targeted and selective to positively modulate problem solving success.

Rather than diminishing the importance of interactivity, our results refine the theory by showing that its benefits depend on a constellation of factors. The influencing factors may include features of the object environment of the experiment, the type of problem and the method of its presentation (see the discussion in Chuderski et al. (2020) on matchstick algebra problems), the individual differences between the solvers such as their cognitive-creative profiles (Salmon and Leikin, 2022), as well as personal characteristics such as shyness (Loginov et al., 2025) and others. Our findings motivate new research: not on whether interactivity helps, but when, how, and for whom interactive engagement with material of the problem supports insight (or outsight).

### 5.2 How the sources of task difficulty and the number of movements influence problem-solving success

In this study, we hypothesized that the impact of motor activity (which we combined across all experiments as a proxy for interactivity) on problem-solving success varies depending on the source of difficulty involved: it will enhance problem-solving performance more when constraints operate at lower levels, and will positively influence the decomposition of chunks, no matter their tightness. We found that solver movements indeed had a different relationship to constraints and chunks, respectively. However, the results painted a more nuanced picture than what we envisioned. While certain problems associated with perceptual chunks (A and D) were not influenced by solver movements, problems with constraints (B and C) showed a *decrease* in the success rate with more movements. Moreover, we have discovered an increase in the total number of movements for the more difficult Problems B, C, and D. Additionally, an increased interactivity of the condition only seemed to elevate the solution rate for Problem A (the success of its solution in Experiments 2 and 3 is significantly higher than in Experiment 1) but led to a decline for Problems B and C (the success of solving them in Experiments 2 and 3 was significantly lower than in Experiment 1).

Several complementary explanations can account for our findings. First, our findings can be explained by the structure of problem solving itself (Weisberg, 2015). When a strategy is unsuccessful or an impasse is reached, it seems reasonable for a solver to try out a different strategy. One such strategy may be using hands to gain new information. Our findings lend tangential support to this claim: in two out of four problems, unsuccessful solvers, on average, performed more movements compared to successful solvers. The greater frequency of movements for more difficult problems may be related to intensive motor-active solution searching, suggesting that greater movement may be a consequence of difficulty, rather than a cause of failure.

Second, the solution of problems with constraints as the sources of difficulty (Problems B and C) may not be related to the solver’s movements at all: they may either have been solved fully or partially through purely mental operations. Moreover, the negative prediction of the number of movements on the success of solving Problems B and C points to the fact that these problems rely more heavily on conceptually driven restructuring, which is not necessarily supported—and may be hindered—by motor activity. The negative influence of the different interactive conditions on performance for the same problems may be explained by the same logic: regardless of whether solvers solve problems with their hands, the interactive material environment drives their solving strategies away from the mental restructuring needed to induce constraint relaxation.

Faced with difficulties and pushed by the instruction to “move the sticks” in the interactive condition, the participants find themselves trapped: by their nature, the operator and tautology constraints cannot be overcome by motor activity. That is, by increasing the number of movements, solvers worsen their position since such a strategy does not make it possible to overcome these sources of difficulty. An analogy to this state of affairs is the well-known experimental fact associated with the nine-dot problem: a verbal prompt does not increase the percentage of correct solutions (Weisberg and Alba, 1981) because it does not affect the sources of difficulty of this task, while special preliminary motor training gives a significant positive effect (Kershaw and Ohlsson, 2004; Spiridonov et al., 2019).

However, this account of the “mental-only” pathway should be regarded as a testable hypothesis for future work, rather than a firm conclusion. Additional explanations also remain viable. Novel VR environments could impose additional handling costs, and instructions emphasizing stick movement may bias solvers toward unproductive strategies. Likewise, interactive conditions may afford exploration that increases physical engagement without improving the representational transformations needed for constraint relaxation. A future sensitivity analysis controlling for time on task would help adjudicate these possibilities in future research.

Overall, we found that neither the movements of the solver nor the interactivity of the condition contribute to the decomposition of the chunk and significantly disrupt the overcoming of the two types of constraints. These results, by proxy, challenge the notion of a universal—i.e., one equally influencing all problems—positive role of interactivity in problem solving. Indeed, the field is moving towards searching for fine-grained accounts of interactive solution processes: “interactivity is not a panacea, it is not a universal degreaser that invariably oils the cognitive cogs” (Vallée-Tourangeau, 2025). This paper is one such account studying how relative problem difficulty plays into solution success in various interactive conditions. Although we were unable to prompt our solvers to move their hands while solving, the negative connection of existing motor activity with the success of solving problems with higher-level constraints points to the fact that movements can not only elevate (as in Weller et al., 2011), but also hinder performance. It opens up a question: what mechanisms can explain the bidirectional effect of motor activity for problems that require the relaxation of constraints that are not directly hinted at by solver movements (as opposed to chunk decomposition)?

The absence of interactive solutions amongst our participants can also explain why we have not seen a pronounced effect of motor activity on performance for problems A and D. Testing our hypotheses with proper interactive solutions is a promising direction for further research.

## 6 Conclusion

This study critically examined the relationship between motor activity and problem-solving success in matchstick algebra problems with varying sources of difficulty. Across three experimental series and multiple interactive conditions, we consistently found that the mere presence of an interactive environment does not guarantee interactive solutions, nor does it lead to improved performance.

This paper’s drive was to specify the broad notion of interactivity. We show that the effectiveness of physical interaction during problem-solving is contingent upon the specific cognitive demands of the problem: while decomposition of perceptual chunks (as in problems A and D) appears unaffected by solver movement, the relaxation of higher-level constraints (as in problems B and C) may actually be *hindered* by increased motor activity. The constraints inherent in these problems—specifically the operator and tautology constraints—seem to be disturbed rather than aided by intensive movements. This observation links back to the idea that certain types of problems, especially those requiring the relaxation of higher-level mental constraints, might not benefit from externalized problem solving strategies.

Future research could fruitfully explore the conditions under which physical engagement with a problem representation aids or impedes performance. This includes identifying the specific movements that align with or diverge from the cognitive operations required to restructure a problem representation. Our study suggests that movements can mislead solvers when motor actions are irrelevant to the underlying constraints. Ultimately, moving from the broad-brush notion of interactivity to fine-grained accounts of how, when, and why people “think with their hands” remains an important direction for future research.

### 6.1 Limitations

A limitation of the present analysis is that the unified Bayesian regression model was not implemented as a fully hierarchical (multilevel) model and therefore did not include participant-level random effects, which may restrict the generalizability of the parameter estimates. In addition, we did not incorporate formal inter-rater reliability metrics to evaluate movement coding; instead, we relied on collective discussions during protocol annotation to achieve an acceptable level of coder agreement.

## Data Availability

The datasets presented in this study can be found in online repositories. The names of the repository/repositories and accession number(s) can be found at: https://osf.io/sdpr3/.
