# Supplementary material for: The modulating role of sources of difficulty in interactive matchstick algebra
Source: Front Psychol. 2026 Jan 19;16:1691864. doi: 10.3389/fpsyg.2025.1691864 (PMC12862482; doi:10.3389/fpsyg.2025.1691864)
Supplement: Supplementary file 1 [file Data_Sheet_1.DOCX]

Supplementary Material

The analysis of the effect of the interactive condition for three minute period (4.4.1.2)

Since the experiment by Weller et al. (2011) limited the time to solve the problem to 3 minutes, we analyzed the results using the first 3 minutes of the solution. The analytical scheme fully corresponds to the analysis of the 5-minute version reported above. As for the 5-minute period, we used weakly informative priors that were placed on all coefficients (Normal(0, 2.5). Four Markov chains were run for 2,000 iterations each, with 1,000 warmup iterations. Convergence diagnostics indicated no issues (all Ȓ < 1.01; no divergent transitions).

We found that — for problem A — in experiment 1, the posterior median for the intercept was 2.3 (95% CrI [1.5, 3.3]), corresponding to a baseline accuracy in the *Static Sticks* condition of approximately 91%. The effect of the *Assembled Sticks* condition was uncertain: the posterior median was -0.27 (95% CrI [-1.57, 1.02]), with Pr(β > 0) = 0.34. This corresponds to a predicted accuracy of approximately 88% (-3 pp), but with substantial uncertainty. The Bayes factor (BF₁₀ = 0.23) provided moderate evidence in favor of the null model relative to the model including condition. However, only 24% of the posterior distribution for the condition coefficient fell within the predefined ROPE (-0.2, 0.2 on the log-odds scale), indicating insufficient posterior mass to claim practical equivalence to zero. Taken together, the credible interval, Bayes factor, and ROPE analysis suggest that the data do not allow a firm conclusion about the presence or the practical absence of a condition effect.

In experiments 2 and 3, problem A was excluded from the analysis since only 4 out of 88 participants in experiment 2, and 2 out of 69 participants in experiment 3, did not solve it successfully.

For problem B — in experiment 1, the posterior median for the intercept was 1.16 (95% CrI [0.54, 1.83]), corresponding to a baseline accuracy in the *Static Sticks* condition of approximately 76%. The effect of the *Assembled Sticks* condition was uncertain: the posterior median was -0.27 (95% CrI [-1.17, 0.6]), with Pr(β > 0) = 0.27. This corresponds to a predicted accuracy of approximately 70% (-6 pp), but with substantial uncertainty. The Bayes factor (BF₁₀ = 0.24) provided moderate evidence in favor of the null model relative to the model including condition. However, only 31% of the posterior distribution for the condition coefficient fell within the predefined ROPE (-0.2, 0.2 on the log-odds scale), indicating insufficient posterior mass to claim practical equivalence to zero. Taken together, the credible interval, Bayes factor, and ROPE analysis suggest that the data do not allow a firm conclusion about the presence or the practical absence of a condition effect.

In Experiment 2, the posterior median for the intercept was 1.59 (95% CrI [0.58, 2.84]), corresponding to a baseline accuracy in the *Static paper* condition of approximately 83%. The effects of *Static Sticks*, *Interactive sticks* and *Interactive assembled sticks* were uncertain: the posterior median was -0.73 (95% CrI [-2.27, 0.76]), -0.87 (95% CrI [-2.41, 0.49]), -0.5 (95% CrI [-1.96, 0.92]), with Pr(β > 0) = 0.17, Pr(β > 0) = 0.10, Pr(β > 0) = 0.26, respectively. This corresponds to a predicted accuracy of approximately 70% (-13 pp), 67% (-16 pp), 75% (-8 pp), respectively, but with substantial uncertainty. The Bayes factor (BF₁₀ = 0.1) provided moderate evidence in favor of the null model relative to the model including condition. However, only 14%, 10% and 18% of the posterior distribution for the condition coefficients, respectively, fell within the predefined ROPE (-0.2, 0.2 on the log-odds scale), indicating insufficient posterior mass to claim practical equivalence to zero. Taken together, the credible interval, Bayes factor, and ROPE analysis suggest that the data do not allow a firm conclusion about the presence or the practical absence of a condition effect.

In Experiment 3, the posterior median for the intercept was 1.55 (95% CrI [0.66, 2.64]), corresponding to a baseline accuracy in the *Static VR* condition of approximately 82%. The effects of *VR Interactive sticks* and *VR Interactive Assembled Sticks* were uncertain: the posterior median was 0.49 (95% CrI [-1.05, 2.21]) and -1.07 (95% CrI [-2.46, 0.26]), with Pr(β > 0) = 0.72 and Pr(β > 0) = 0.05, respectively. This corresponds to a predicted accuracy of approximately 88% (+6 pp) and 62% (-20 pp), respectively, but with substantial uncertainty. The Bayes factor (BF₁₀ = 0.7) provided weak evidence in favor of the null model relative to the model including condition. However, only 18% and 7% of the posterior distribution for the condition coefficients, respectively, fell within the predefined ROPE (-0.2, 0.2 on the log-odds scale), indicating insufficient posterior mass to claim practical equivalence to zero. Taken together, the credible interval, Bayes factor, and ROPE analysis suggest that the data do not allow a firm conclusion about the presence or the practical absence of a condition effect.

For problem С — in experiment 1, the posterior median for the intercept was 0.14 (95% CrI [-0.38, 0.68]), corresponding to a baseline accuracy in the *Static Sticks* condition of approximately 53%. The effect of the *Assembled Sticks* condition was uncertain: the posterior median was -0.19 (95% CrI [-0.94, 0.58]), with Pr(β > 0) = 0.32. This corresponds to a predicted accuracy of approximately 49% (-4 pp), but with substantial uncertainty. The Bayes factor (BF₁₀ = 0.23) provided moderate evidence in favor of the null model relative to the model including condition. However, only 37% of the posterior distribution for the condition coefficient fell within the predefined ROPE (-0.2, 0.2 on the log-odds scale), indicating insufficient posterior mass to claim practical equivalence to zero. Taken together, the credible interval, Bayes factor, and ROPE analysis suggest that the data do not allow a firm conclusion about the presence or the practical absence of a condition effect.

In Experiment 2, the posterior median for the intercept was -0.62 (95% CrI [-1.55, 0.23]), corresponding to a baseline accuracy in the *Static paper* condition of approximately 35%. The effects of *Static Sticks, Interactive sticks* and *Interactive assembled sticks* were uncertain: the posterior median was -0.81 (95% CrI [-2.37, 0.57]), 0.14 (95% CrI [-1.13, 1.389]), 0.62 (95% CrI [-0.57, 0.82]), with Pr(β > 0) = 0.13, Pr(β > 0) = 0.58, Pr(β > 0) = 0.84, respectively. This corresponds to a predicted accuracy of approximately 19% (-16 pp), 38% (+3 pp), 50% (+15 pp), respectively, but with substantial uncertainty. The Bayes factor (BF₁₀ = 0.29) provided moderate evidence in favor of the null model relative to the model including condition. However, only 12%, 25% and 16% of the posterior distribution for the condition coefficients, respectively, fell within the predefined ROPE (-0.2, 0.2 on the log-odds scale), indicating insufficient posterior mass to claim practical equivalence to zero. Taken together, the credible interval, Bayes factor, and ROPE analysis suggest that the data do not allow a firm conclusion about the presence or the practical absence of a condition effect.

In Experiment 3, the posterior median for the intercept was -0.16 (95% CrI [-0.91, 0.61]), corresponding to a baseline accuracy in the *Static VR* condition of approximately 46%. The effects of *VR Interactive sticks* and *VR Interactive Assembled Sticks* were uncertain: the posterior median was -0.18 (95% CrI [-1.36, 0.91]) and 0.27 (95% CrI [-0.93, 1.38]), with Pr(β > 0) = 0.38 and Pr(β > 0) = 0.68, respectively. This corresponds to a predicted accuracy of approximately 42% (-4 pp) and 53% (+7 pp), respectively, but with substantial uncertainty. The Bayes factor (BF₁₀ = 0.14) provided moderate evidence in favor of the null model relative to the model including condition. However, only 27% and 25% of the posterior distribution for the condition coefficients, respectively, fell within the predefined ROPE (-0.2, 0.2 on the log-odds scale), indicating insufficient posterior mass to claim practical equivalence to zero. Taken together, the credible interval, Bayes factor, and ROPE analysis suggest that the data do not allow a firm conclusion about the presence or the practical absence of a condition effect.

For problem D — in experiment 1, the posterior median for the intercept was 0.45 (95% CrI [-0.09, 1.01]), corresponding to a baseline accuracy in the *Static Sticks* condition of approximately 61%. The effect of the *Assembled Sticks* condition was uncertain: the posterior median was 0.06 (95% CrI [-0.75, 0.89]), with Pr(β > 0) = 0.56. This corresponds to a predicted accuracy of approximately 62% (+1 pp), but with substantial uncertainty. The Bayes factor (BF₁₀ = 0.21) provided moderate evidence in favor of the null model relative to the model including condition. However, only 39% of the posterior distribution for the condition coefficient fell within the predefined ROPE (-0.2, 0.2 on the log-odds scale), indicating insufficient posterior mass to claim practical equivalence to zero. Taken together, the credible interval, Bayes factor, and ROPE analysis suggest that the data do not allow a firm conclusion about the presence or the practical absence of a condition effect.

In Experiment 2, the posterior median for the intercept was 0.85 (95% CrI [-0.01, 1.77]), corresponding to a baseline accuracy in the *Static paper* condition of approximately 70%. The effects of *Static Sticks*, *Interactive sticks* and *Interactive assembled sticks* were uncertain: the posterior median was 0.59 (95% CrI [-0.78, 2.8]), 0.34 (95% CrI [-0.98, 1.76), -0.69 (95% CrI [-1.88, 0.44]), with Pr(β > 0) = 0.78, Pr(β > 0) = 0.69, Pr(β > 0) = 0.13, respectively. This corresponds to a predicted accuracy of approximately 80% (+10 pp), 77% (+7 pp), 54% (-16 pp), respectively, but with substantial uncertainty. The Bayes factor (BF₁₀ = 0.27) provided moderate evidence in favor of the null model relative to the model including condition. However, only 18%, 22% and 16% of the posterior distribution for the condition coefficients, respectively, fell within the predefined ROPE (-0.2, 0.2 on the log-odds scale), indicating insufficient posterior mass to claim practical equivalence to zero. Taken together, the credible interval, Bayes factor, and ROPE analysis suggest that the data do not allow a firm conclusion about the presence or the practical absence of a condition effect.

In Experiment 3, the posterior median for the intercept was 0.78 (95% CrI [0.02, 1.57]), corresponding to a baseline accuracy in the *Static VR* condition of approximately 69%. The effects of *VR Interactive sticks* and *VR Interactive Assembled Sticks* were uncertain: the posterior median was 0.89 (95% CrI [-0.38, 2.45]) and 0.73 (95% CrI [-0.58, 2.24]), with Pr(β > 0) = 0.92 and Pr(β > 0) = 0.85, respectively. This corresponds to a predicted accuracy of approximately 84% (+15 pp) and 82% (+13 pp), respectively, but with substantial uncertainty. The Bayes factor (BF₁₀ = 0.26) provided moderate evidence in favor of the null model relative to the model including condition. However, only 11% and 14% of the posterior distribution for the condition coefficients, respectively, fell within the predefined ROPE (-0.2, 0.2 on the log-odds scale), indicating insufficient posterior mass to claim practical equivalence to zero. Taken together, the credible interval, Bayes factor, and ROPE analysis suggest that the data do not allow a firm conclusion about the presence or the practical absence of a condition effect.
